# Capsid and Genome Modification Strategies to Reduce the Immunogenicity of Adenoviral Vectors

**DOI:** 10.3390/ijms22052417

**Published:** 2021-02-28

**Authors:** Florian Kreppel, Claudia Hagedorn

**Affiliations:** Center for Biomedical Education and Research (ZBAF), Witten/Herdecke University (UW/H), Stockumer Str. 10, 58448 Witten, Germany

**Keywords:** adenovirus, vector, PEGylation, HPMA, immune response, shielding, stealthing, cloaking

## Abstract

Adenovirus-based gene transfer vectors are the most frequently used vector type in gene therapy clinical trials to date, and they play an important role as genetic vaccine candidates during the ongoing SARS-CoV-2 pandemic. Immediately upon delivery, adenovirus-based vectors exhibit multiple complex vector-host interactions and induce innate and adaptive immune responses. This can severely limit their safety and efficacy, particularly after delivery through the blood stream. In this review article we summarize two strategies to modulate Ad vector-induced immune responses: extensive genomic and chemical capsid modifications. Both strategies have shown beneficial effects in a number of preclinical studies while potential synergistic effects warrant further investigations.

## 1. Introduction

While viruses have been evolutionary optimized to deliver their genes into host target cells with high specificity and efficiency, the hosts have developed complex defense mechanisms that limit or prevent such infections. To date, the most efficient gene transfer vectors are derived from viruses. As a consequence, to achieve safe and efficacious in vivo gene delivery by virus-based vectors a comprehensive understanding of the molecular mechanisms underlying antiviral immune reactions is mandatory. In addition, tools are needed to modify vectors appropriately with the aim to either prevent or exploit such immune reactions. The immune system is a complex and interwoven network with numerous different cell types and noncellular components being involved. It is divided into an innate and an adaptive arm. The innate immune response relies on a limited number of proteins and reacts within seconds or minutes after an infectious event. Innate immune responses are not antigen-specific and do not result in immunological memory. However, innate immune sensing creates an inflammatory environment that conditions adaptive immune responses. The adaptive parts of the immune system rely on clonal expansion of antigen-specific B- and T-cells and generate an immunological memory. Sensing of viral structures (e.g., capsid proteins) by the innate immune system may provoke tissue invasion of innate immune cells and release of inflammatory chemokines and cytokines. These events induce an inflammatory (and anti-viral) state in the respective tissue, resulting in decreased transduction and potential activation of adaptive immune responses. Activation of dendritic cells (DCs) and subsequent antigen-presentation is a critical step linking innate to adaptive immune responses.

Adenoviruses (Ad) are non-enveloped, icosahedral viruses with a ~35 kb linear double-stranded (ds) DNA genome [1,2]. They can infect many cell types including low-proliferative or quiescent cell populations and antigen-presenting cells (APC). The results of intensive research over decades now allow for controlled and defined modifications of the viral genome resulting in stable recombinant Ad vectors with a large cloning capacity (up to 36 kb). Moreover, Ad vectors can be produced on a large scale (>1 × 10^12^ particles/mL) while meeting clinical good manufacturing practice (GMP) standards. Given these numerous advantages Ad-derived vectors are currently the most commonly used vectors in gene therapy, cancer and vaccine clinical trials worldwide (http://www.abedia.com/wiley/vectors.php (accessed on 10 January 2021)). The first gene therapy/oncolytic products based on Ad were released already eighteen years ago [3] and, importantly, adenovirus vectors play an important role as genetic vaccines (and vaccine candidates) during the ongoing SARS-CoV-2 pandemic. In this review we will focus on two promising strategies to modulate Ad vector-induced immune responses: genetic approaches and chemical shielding. If not stated otherwise our presentation refers to human adenovirus type five. This is one of the best characterized adenovirus types, and, while its clinical applicability may still be limited, it significantly contributes to the fact that Ad vectors are an enduring toolbox for basic and applied research. 

## 2. Adenovirus vector types based on human adenovirus type five 

So-called first-generation (FG) vectors based on human adenovirus type five have a transgene capacity of up to 8 kilobases (kb) due to deletion of the E1 and/or E3 gene regions. These vectors exhibit strong short-term transgene expression in immune-competent individuals due to leaky expression of viral genes that triggers vector-directed immune responses. Viral gene products, and in some cases the transgene itself, influence cellular immune responses leading to extinction of gene expression [4,5]. Therefore, these vectors have great potential to be utilized as genetic vaccines. Additional deletion of E2 and/or E4 gene region (second-generation vectors) led to reduced viral gene expression [6]. This results in not only in reduced inflammatory responses [7,8,9] but also higher transgene capacity (up to 12 kb) and prolonged transgene expression [10]. A third generation of Ad vectors, devoid of all viral genes, have a cargo capacity of 36 kb. These so-called high-capacity (HC-Ad) or helper-dependent (HD-Ad) vectors exhibit low immunotoxicity and stable long-term transgene expression in several tissues (reviewed in [11]). However, despite the progress that has been made in successfully manipulating the adenoviral genome (see the following paragraphs), the safe and efficient application of Ad vectors is still limited and faces several obstacles. The harshest drawback in gene therapy was the death of a patient in a clinical trial in 1999. High doses of a second-generation Ad vector triggered strong inflammatory reactions and finally resulted in multi organ failure [12]. This incident demonstrated very early the need of a thorough understanding of host—vector interactions as a prerequisite for safe Ad-mediated gene therapy in humans.

## 3. Immune responses to Ad vectors

Systemic Ad vector administration for gene transfer purposes has been shown to result in quick vector sequestration from the blood stream. In mice an almost complete elimination of virus particles from the blood occurs within 30 min, with a half-life of less than 2 min [13]. The removal of Ad particles coincides with a release of proinflammatory factors such interleukin (IL)-6, tumor necrosis factor α (TNF α), IL-12, RANTES, and interferon γ induced protein 10 [14,15,16,17,18,19]. Most probably, the majority of the released chemokines and cytokines are produced in the spleen, whereas macrophages and dendritic cells (DCs) from the spleen have been shown to be key effector cells in innate immune response. Activation of innate immunity has been observed to be dose-dependent in rodents and nonhuman primates [15]. Administration of high Ad vector doses (up to 2 × 10^12^ vector particles) in human gene therapy trials led to elevated serum level of IL-6 und IL-1 [20,21,22]. Of note, data suggest that activation of innate immunity is largely independent of the transduction process itself: inactivated vector particles activated the innate immunity in a similar manner as biologically active particles [15]. Moreover, an acute inflammatory response was observed in nonhuman primates after high dose administration of a HD-Ad vector lacking all viral genes [23]. These observations suggest that activation of innate immunity rather relates to the Ad vector capsid proteins themselves than to viral gene expression. Importantly, the activation of complement system significantly contributes to activation of innate immune response [24]. Systemically administered Ad vectors activate the complement system via the classical pathway [25] as well as direct interaction of C3 with the capsid [26]. Of note, binding of complement factors to the capsid can also compete with binding of blood coagulation factor X (FX) [27]. Capsid-bound FX leads to sequestration of vectors in hepatocytes.

Ads and Ad-derived vectors are highly immunogenetic, and because human Ads (hAds) are ubiquitous, the serum prevalence to one or more types is nearly universal [28]. Therefore, the majority of humans have circulating antibodies against the common Ad types (including but not limited to Ad5) and infected individuals develop prolonged immunity to the virus [29]. The three structure-determining viral capsid protein are hexon, fiber and penton base [30]. Among those the most abundant capsid protein hexon contains so called hypervariable regions (HVR) which are type-specific [31] and are considered to harbor major immune determinants. Hence, anti-Ad antibodies predominantly target hexon [32,33,34,35], but may also be directed against penton [36] and fiber [33]. Circulating anti-Ad antibodies have an immediate impact on the therapeutic efficiency: besides significantly attenuating vector-mediated gene transfer efficiency [37], they also have been shown to worsen vector toxicity [38,39]. Bound to the viral capsid, antibodies mediate sequestration by Fc receptor (FcR)-positive cells including DCs [40], neutrophils [41], and tissue-resident macrophages [42], resulting in vector clearance and poor tissue transduction. The presence of pre-existing anti-Ad antibodies appears to stimulate a stronger innate immune response [42], rising serious concerns when high vector doses or sequential administration is needed. Besides the effects of neutralizing antibodies, Ad-specific T cells also contribute to the fading efficacy of Ad-derived vectors in individuals with pre-existing immunity [4]. Memory Ad-specific CD8+ and CD4+ T-cells have been shown to be responsible for elimination of target cells that express viral and transgene products [43,44,45].

## 4. Vector genome modifications to dampen immune responses

The construction of third-generation Ad vectors, also called helper-dependent (HD-Ad) or high-capacity (HC-Ad) Ad vectors, is based on extensive genetic modifications up to the deletion of all viral genes except for cis-acting elements needed for genome replication (ITR) and packaging (ψ). These global deletions offer high cloning capacity (~36 kb; high-capacity Ad vectors, HD-Ad) and enable delivery of multiple transgenes, complete genomic loci or at least large regulatory sequences to regulate e.g., tissue-specific transgene expression. Due to the lack of viral gene expression, HD-Ad exhibit prolonged transgene expression and reduced toxicity. For efficient DNA packaging a minimum vector size of 27 kb should be maintained [46,47], so deletions must be replaced by so-called stuffer DNA. First DNA stuffers originated from lambda phage, bacterial or yeast DNA [47]. Injection of lambda-stuffed HD-Ad in mice, however, resulted in only transient gene expression and cytotoxic T-cell (CTL) responses against peptides that were encoded by lambda stuffer DNA. Replacing the lambda DNA with intronic sequences from human *hypoxanthine-guanine phosphoribosyltransferase* (*HPRT*) gene resulted in prolonged gene expression and no induction of a CTL response was detected [48]. When choosing stuffer DNA and designing transgene expression cassettes, the intracellular part of innate immunity should also be considered. Toll-like receptors (TLR) are pattern recognition receptors of pathogen-derived ligands [49,50,51]. Four of ten known TLRs are described to recognize nucleic acids. Residing in endosomes or lysosomes, TLR9 detects dsDNA and can be activated by unmethylated CpG motifs [52,53]. Irrespective of encoding expression cassettes, HD-Ad were shown to activate TLR9 signaling in primary macrophages, indicating the impact of stuffer DNA on innate immunity. Moreover, TLR9 antagonists attenuated the acute inflammatory response in vivo [54].

In the following we will exemplarily highlight few promising studies addressing long-term transgene expression and immunogenicity of HD-Ad.

For treatment of hypercholesteremia in apolipoprotein E (apoE) deficient mice a first-generation Ad vector (FG-Ad) encoding the *apoE* cDNA as well as a HD-Ad harboring the genomic *apoE* locus were compared. After intravenous injection, FG-Ad resulted in transiently (28 days) decreased cholesterol serum level within normal range. In contrast, delivery of the *apoE* genomic locus using HD-Ad yielded stable reduction of cholesterol serum level for up to two and a half years. In addition, only FG-Ad induced elevated serum level of liver enzymes aspartate transaminase (AST) and alanine transaminase (ALT) indicating hepatoxicity, whereas there was no such evidence for HD-Ad. However, both vectors induced production of Ad-specific antibodies hindering re-administration [55]. Another study in rodents used HD-Ad to achieve liver-restricted expression of uridine diphospho-glucuronosyl transferase 1A1 (UGT1A1), an enzyme that is required for glucuronidation of bilirubin. A lack of UGT1A1 results in persistent unconjugated hyperbilirubinemia as seen in Crigler–Najjar (CN) syndrome. Systemic single-dose administration of HD-Ad encoding for UTG1A1 resulted in reduction of plasma bilirubin levels for more than 2 years. No acute liver toxicity and only transient thrombocytopenia was detected [56].

Similar findings were observed in clinically relevant large animal models. Liver-directed, HD-Ad-mediated expression of human α1-antitrypsin (hAAT) in baboons exhibited prolonged hAAT expression for > 1 year in two out of three animals. The third animal, however, had significantly lower hAAT serum levels due to antibody development targeting the hAAT protein. In contrast, when FG-Ad was used for hAAT delivery, transgene expression lasted for only 3–5 month. Here this was not due to antibody formation against hAAT, but development of Ad-specific CD4+ T-cells, resulting in the elimination of all transduced liver cells. A phenomenon that was not observed for HD-Ad [57]. One very remarkable study in terms of long-term gene expression demonstrated liver-restricted transgene expression for up to seven years in nonhuman primates after a single-dose administration of HD-Ad. Despite this success, authors observed a slow but steady decline in transgene expression that is probably due to a gradual loss of transduced cells because of physiologic liver turnover [58], highlighting a central problem in gene therapy. A life-long transgene expression in humans basically relies on two options; either (i) replicating, though preferably non-integrating, vector systems; or (ii) efficient vector re-administration, the latter being hampered by the development of anti-vector neutralizing antibodies (NABs) [58]. Despite the advantages of HD-Ad over FG-Ad, the viral capsid is identical and could induce acute inflammatory responses [12,23,59]. This acute toxicity is dose-dependent and, as discussed above, characterized by elevated serum level of proinflammatory cytokines as a consequence of innate immune system activation. Low vector doses result in low toxicity but unfortunately also in low tissue transduction. Vector doses required for efficient transduction however are accompanied with acute toxicity that increases with vector-doses. Shielding of the viral capsid may therefore help to overcome both fundamental obstacles, acute inflammatory response and development of neutralizing antibodies.

## 5. Chemical shielding to reduce unwanted surface interactions

An elegant method to prevent viral vectors from being recognized by both, innate and adaptive immune cells, is to equip the viral capsid with a “magic cap”. Pharmaceutical studies have shown before that polyethylene glycol (PEG) reduces antigenicity and immunogenicity of therapeutic protein compounds [60,61]. Thus, coupling capsid proteins to polymers such as PEG paved the way for various shielding strategies that are discussed below.

Due to the hydrophilic nature of covalently attached polymers like PEG and poly-N-(2-hydroxypropyl)methacrylamide (pHPMA), another important shielding moiety [62], polymer-modified vector particles in solution are surrounded by a stable water shell. The polymer molecules and their water shells shield the vectors particles from undesired vector-host-interactions by steric hindrance and, thus, reduce immune cell recognition. The chemical modifications are performed after production and purification of the virus avoiding the need of specific producer cell lines. Usually, polymers are coupled to ε-amine groups from lysine side groups that are randomly distributed on the capsid surface. By amine-specific PEGylation the vast majority of the 18,000 solvent-exposed lysine residues on the surface of an Ad5-based vector particle can be PEGylated so that a dense PEG shield is generated. Consistently, when performed for the first time in 1999, O’Riordan *et al.* demonstrated that PEGylated vector particles evaded neutralization both, in vitro by purified antihexon antibodies and in pre-immunized mice in vivo [63]. Croyle et al. analyzed Ad-specific CTL response and development of anti-Ad NABs after single-dose administration of PEGylated Ad vectors in mice. Consistent with findings of O’Riordan *et al.*, PEGylated Ads mediated prolonged transgene expression and exhibited a decreased induction of Ad-specific CTLs. Analysis of cytokine profile revealed reduced Th1 responses but no difference in Th2 responses. However, Ad vector PEGylation still stimulated development anti-Ad NABs, albeit to a low extent, resulting in reduced transgene expression after sequential vector administration [64]. In a continuative study, Croyle et al. analyzed transgene expression in mice pre-immunized with native unshielded Ad. Despite high titers of neutralizing anti-Ad antibodies, delivery of PEGylated first-generation vectors resulted in significant transgene expression in the liver, although expression level were lower than those in animals who received a single-dose of unmodified Ad without pre-immunization. Further, duration of transgene expression was only slightly longer when PEGylated Ad vectors were delivered [65]. These findings highlight the role of newly synthesized viral and/or transgenic proteins in the elimination of transgene expression, as they are unaffected by PEGylation. However, PEGylation of Ad vectors not only prevents neutralization by pre-existing antibodies, but also association with Kupffer cells in vivo. Corroborating in vitro experiments using a murine macrophage cell line further demonstrated decreased IL-6 production upon incubation with PEGylated vectors and decreased vector particle uptake [66]. Outlining the importance of vector sequestration by macrophages for induction of innate immune responses, these findings suggest that PEGylation of Ad vectors might be valuable tool to alter vector interaction with innate immune cells, in particular macrophages.

After coupling, nonreactive ends of monovalent PEGs protrude from the capsid surface, while multivalent pHPMA molecules are linked to numerous reactive sites coupling the polymer to the viral capsid. Capsid shielding using pHPMA prevented vector particles from antibody neutralization [62] and binding to blood components [67], and resulted in prolonged blood circulation in vivo [68]. However, despite these beneficial effects, amine-directed polymer coupling is associated with some limitations. To achieve the above described benefits, large polymer moieties are required that might impair virus bioactivity. Further, because amine-directed coupling occurs randomly throughout the whole vector capsid surface, this approach does not allow for position or motif-specific shielding. Finally, polymer-modified vector particles show high heterogeneity even within one preparation.

A genetichemical concept for vector shielding overcoming some of these limitations was introduced by Kreppel et al. This concept based on the genetic introduction of cysteines in the capsid at solvent-exposed positions like fiber HI-loop [69], protein IX [70], and hexon hypervariable regions [71,72]. Although not naturally occurring, surface cystein-bearing Ad vectors are producible at high titers in conventional producer cells. The introduced cysteines allow specific coupling with thiol group-reactive moieties not only on certain capsomers, but also at different positions within a certain capsomer. The geneti-chemical approach allows one to study vector-host interactions in a position-specific manner and has been shown to overcome numerous obstacles for Ad vector design. Being the most abundant capsid protein, hexon is involved in many undesired interactions (e.g., neutralizing antibodies, FX-binding). Therefore, thiol-based modification strategies were so far predominantly applied to hexon. Coupling certain PEG moieties to HVR5 of hexon prevented Ad vector particles to interact with FX [71,73]. Ad vector particles carrying mutations in the fiber knob to inhibit binding to its natural receptor (CAR) and in HVR7 to prevent binding of FX, and being additionally equipped with a cysteine in HVR1 for position-specific PEGylation, were shown to evade antibody- and complement-mediated neutralization as well as scavenger receptor-mediated uptake, without loss of infectivity [72]. However, covalent shielding does have an impact on intracellular trafficking processes. Indeed, polymer coupling to the capsid surface via degradable disulfide-bonds resulted in higher infectivity compared to vector particles that were covalently coupled to a polymer [67]. Comparing irreversible versus bioresponsive shields based on pHPMA revealed that neither the mode of shielding nor co-polymer charge had an impact on cell entry but affected particle trafficking to the nucleus. Employing a bioresponsive shield with positively charged pHPMA co-polymers allowed for particle trafficking to the nucleus maintaining the high transduction efficiencies of Ad vectors in vitro and in vivo [74]. Most recently, a ‘stealth’ layer based on a hexon-binding single-chain antibody variable fragment (scFv) was successfully used to shield vector particles from neutralizing antibodies in vitro while maintaining its infectivity [75].

So far, these data indicate that shielding of viral capsids is a powerful tool to prevent interactions of the immune system with Ad vectors, circumventing antibody mediated neutralization and mitigating acute toxicity. Chemical capsid modifications of Ad vectors in general need to be performed in a way that does not interfere with intracellular trafficking of the vector particles or prevents particle disassembly after uptake into the target cells [70,76,77]. There are, however, applications such as vaccination where a purposeful activation of cellular and humoral immune responses are desired.

## 6. Exploiting adenoviral immunogenicity in vaccine development

As described above, Ads are highly immunogenic and can induce both, innate and adaptive immune responses. While this may lead to undesired side-effects in gene replacement strategies, these effects can be exploited for vaccination purposes. A successful vaccination strategy requires the induction of a cytokine profile orchestrating the maturation of B- and cytotoxic T-cells, resulting in a prolonged and boostable immune response. Naturally, Ad vectors cause lytic infections resulting in short antigen presentation time of individually infected cells, thereby potentially favoring a CD4+ immune response. Most Ad vectors currently used for vaccination in clinical and pre-clinical trials are FG-Ad (reviewed for example in [78]) and benefit from strong immune responses due to leaky expression of viral genes that contributes to the intrinsic adjuvant Ad vector immunogenicity. The current SARS-CoV-2 pandemic boosted the need of genetic vaccines and Ad-based vectors encoding the SARS-CoV-2 spike protein are advanced candidates [79,80,81,82,83]. In a clinical phase 2 trial a FG-Ad based vaccine has been shown to induce humoral and cellular immune response against the receptor binding domain (RBD) of SARS-CoV-2 spike protein [80]. However, transgene and viral gene products are presented competitively by MHC I and MHC II molecules, resulting in anti-Ad CTLs that recognize and eliminate Ad-transduced cells [84,85]. In terms of a strong anti-transgene immune response, it has been shown that HD-Ad might be superior to FG-Ad. Due to prolonged antigen expression and the lack of any viral gene expression, HD-Ad have been shown to induce strong anti-transgene T-cell and antibody responses [86,87,88,89] and resulted in lower tissue damage [88]. Additionally, NABs are a significant obstacle in both HD-Ad and FG-Ad vectored vaccination. Pre-existing NABs against one or more Ad types can be found in nearly all humans [28], and antibodies are generated with each vector administration. The combination of anti-Ad NABs and anti-Ad CTL has significant potential to impair transgene expression and generation of anti-transgene CTL.

Because shielding vector capsids with polymers such as PEG has been shown to evade recognition by innate immune cells, prevents vector particles from being trapped by Kupffer cells and macrophages, and protects from neutralizing antibodies [63,65,66], it might also be employable in Ad-mediated vaccination. Investigating the effect of Ad5 capsid PEGylation on antibody and T cell responses in mice, it was demonstrated PEGylated vectors are capable to efficiently induce both, humoral and cellular immune responses [90]. Also, PEGylated vectors enabled significant boosting in pre-immunized mice. Further, priming with PEGylated vectors enabled both, PEGylated and unmodified vectors to produce stronger immune responses [91]. However, both studies applied amine-directed polymer coupling and subsequently observed reduced infectivity of PEGylated vectors. Hence, new strategies in Ad-based vaccine development should consider position-specific shielding of selected capsomers. This might help to balance the need of maintaining particle infectivity while protecting from neutralizing antibodies.

## 7. Conclusions

Adenovirus vectors belong to the most immunogenic vectors. Thus, unwanted immune reactions need to be dampened and controlled in order to enable safe Ad vector-mediated gene delivery. Two major strategies have been employed for Ad vectors: (i) genome modifications that delete all viral genes to prevent immune reactions due to leaky virus gene expression from the vectors and (ii) capsid shielding strategies based on chemical capsid modifications. While Ad vectors devoid of all viral coding sequences have shown prolonged gene expression in animal models, chemically “stealthed” vectors exhibited dampened innate immune responses. By combining both approaches with constant results from the discovery of additional rare Ad types and Ad species, it appears very likely that improved gene transfer vectors, Ad-based genetic vaccines and oncolytics will be developed in the near future. Because Ad vector-based vaccines are currently an important tool to fight the COVID-19 pandemic, it might become of even higher relevance to develop novel strategies to circumvent pre-existing antivector immunity.

## References

[1] Reddy V.S., Natchiar S.K., Stewart P.L., Nemerow G.R. (2010). Crystal structure of human adenovirus at 3.5 A resolution. Science.

[2] Liu H., Jin L., Koh S.B.S., Atanasov I., Schein S., Wu L. (2010). Atomic structure of human adenovirus by cryo-EM reveals interactions among protein networks. Science.

[3] Pearson S., Jia H., Kandachi K. (2004). China approves first gene therapy. Nat. Biotechnol..

[4] Yang Y., Nunes F.A., Berencsi K., Furth E.E., Gönczöl E., Wilson J.M. (1994). Cellular immunity to viral antigens limits E1-deleted adenoviruses for gene therapy. Proc. Natl. Acad. Sci. USA.

[5] Yang Y., Ertl H.C., Wilson J.M. (1994). MHC class I-restricted cytotoxic T lymphocytes to viral antigens destroy hepatocytes in mice infected with E1-deleted recombinant adenoviruses. Immunity.

[6] Amalfitano A., Hauser M.A., Hu H., Serra D., Begy C.R., Chamberlain J.S. (1998). Production and characterization of improved adenovirus vectors with the E1, E2b, and E3 genes deleted. J. Virol..

[7] Engelhardt J.F., Ye X., Doranz B., Wilson J.M. (1994). Ablation of E2A in recombinant adenoviruses improves transgene persistence and decreases inflammatory response in mouse liver. Proc. Natl. Acad. Sci. USA.

[8] Gao G.P., Yang Y., Wilson J.M. (1996). Biology of adenovirus vectors with E1 and E4 deletions for liver-directed gene therapy. J. Virol..

[9] Wang Q., Greenburg G., Bunch D., Farson D., Finer M.H. (1997). Persistent transgene expression in mouse liver following in vivo gene transfer with a delta E1/delta E4 adenovirus vector. Gene Ther..

[10] Armentano D., Zabner J., Sacks C., Sookdeo C.C., Smith M.P., St George J.A. (1997). Effect of the E4 region on the persistence of transgene expression from adenovirus vectors. J. Virol..

[11] Rosewell A., Vetrini F., Ng P. (2011). Helper-Dependent Adenoviral Vectors. J. Genet. Syndr. Gene Ther..

[12] Raper S.E., Chirmule N., Lee F.S., Wivel N.A., Bagg A., Gao G. (2003). Fatal systemic inflammatory response syndrome in a ornithine transcarbamylase deficient patient following adenoviral gene transfer. Mol. Genet. Metab..

[13] Alemany R., Suzuki K., Curiel D.T. (2000). Blood clearance rates of adenovirus type 5 in mice. J. Gen. Virol..

[14] Muruve D.A., Barnes M.J., Stillman I.E., Libermann T.A. (1999). Adenoviral gene therapy leads to rapid induction of multiple chemokines and acute neutrophil-dependent hepatic injury in vivo. Hum. Gene Ther..

[15] Schnell M.A., Zhang Y., Tazelaar J., Gao G.P., Yu Q.C., Qian R. (2001). Activation of innate immunity in nonhuman primates following intraportal administration of adenoviral vectors. Mol. Ther. J. Am. Soc. Gene Ther..

[16] Zhang Y., Chirmule N., Gao G.P., Qian R., Croyle M., Joshi B. (2001). Acute cytokine response to systemic adenoviral vectors in mice is mediated by dendritic cells and macrophages. Mol. Ther. J. Am. Soc. Gene Ther..

[17] Lieber A., He C.Y., Meuse L., Schowalter D., Kirillova I., Winther B. (1997). The role of Kupffer cell activation and viral gene expression in early liver toxicity after infusion of recombinant adenovirus vectors. J. Virol..

[18] Borgland S.L., Bowen G.P., Wong N.C., Libermann T.A., Muruve D.A. (2000). Adenovirus vector-induced expression of the C-X-C chemokine IP-10 is mediated through capsid-dependent activation of NF-kappaB. J. Virol..

[19] Bowen G.P., Borgland S.L., Lam M., Libermann T.A., Wong N.C.W., Muruve D.A. (2002). Adenovirus vector-induced inflammation: Capsid-dependent induction of the C-C chemokine RANTES requires NF-kappa B. Hum. Gene Ther..

[20] Reid T., Galanis E., Abbruzzese J., Sze D., Andrews J., Romel L. (2001). Intra-arterial administration of a replication-selective adenovirus (dl1520) in patients with colorectal carcinoma metastatic to the liver: A phase I trial. Gene Ther..

[21] Reid T., Galanis E., Abbruzzese J., Sze D., Wein L.M., Andrews J. (2002). Hepatic arterial infusion of a replication-selective oncolytic adenovirus (dl1520): Phase II viral, immunologic, and clinical endpoints. Cancer Res..

[22] Ben-Gary H., McKinney R.L., Rosengart T., Lesser M.L., Crystal R.G. (2002). Systemic interleukin-6 responses following administration of adenovirus gene transfer vectors to humans by different routes. Mol. Ther. J. Am. Soc. Gene Ther..

[23] Brunetti-Pierri N., Palmer D.J., Beaudet A.L., Carey K.D., Finegold M., Ng P. (2004). Acute toxicity after high-dose systemic injection of helper-dependent adenoviral vectors into nonhuman primates. Hum. Gene Ther..

[24] Kiang A., Hartman Z.C., Everett R.S., Serra D., Jiang H., Frank M.M. (2006). Multiple innate inflammatory responses induced after systemic adenovirus vector delivery depend on a functional complement system. Mol. Ther. J. Am. Soc. Gene Ther..

[25] Cichon G., Boeckh-Herwig S., Kuemin D., Hoffmann C., Schmidt H.H., Wehnes E. (2003). Titer determination of Ad5 in blood: A cautionary note. Gene Ther..

[26] Jiang H., Wang Z., Serra D., Frank M.M., Amalfitano A. (2004). Recombinant adenovirus vectors activate the alternative complement pathway, leading to the binding of human complement protein C3 independent of anti-ad antibodies. Mol. Ther. J. Am. Soc. Gene Ther..

[27] Xu Z., Qiu Q., Tian J., Smith J.S., Conenello G.M., Morita T. (2013). Coagulation factor X shields adenovirus type 5 from attack by natural antibodies and complement. Nat. Med..

[28] Abbink P., Lemckert A.A.C., Ewald B.A., Lynch D.M., Denholtz M., Smits S. (2007). Comparative Seroprevalence and Immunogenicity of Six Rare Serotype Recombinant Adenovirus Vaccine Vectors from Subgroups B and D. J. Virol..

[29] Schmitz H., Wigand R., Heinrich W. (1983). Worldwide epidemiology of human adenovirus infections. Am. J. Epidemiol..

[30] Benevento M., Di Palma S., Snijder J., Moyer C.L., Reddy V.S., Nemerow G.R. (2014). Adenovirus composition, proteolysis, and disassembly studied by in-depth qualitative and quantitative proteomics. J. Biol. Chem..

[31] Rux J.J., Kuser P.R., Burnett R.M. (2003). Structural and phylogenetic analysis of adenovirus hexons by use of high-resolution x-ray crystallographic, molecular modeling, and sequence-based methods. J. Virol..

[32] Toogood C.I., Crompton J., Hay R.T. (1992). Antipeptide antisera define neutralizing epitopes on the adenovirus hexon. J. Gen. Virol..

[33] Bradley R.R., Lynch D.M., Iampietro M.J., Borducchi E.N., Barouch D.H. (2012). Adenovirus serotype 5 neutralizing antibodies target both hexon and fiber following vaccination and natural infection. J. Virol..

[34] Qiu H., Li X., Tian X., Zhou Z., Xing K., Li H. (2012). Serotype-Specific Neutralizing Antibody Epitopes of Human Adenovirus Type 3 (HAdV-3) and HAdV-7 Reside in Multiple Hexon Hypervariable Regions. J. Virol..

[35] Sumida S.M., Truitt D.M., Lemckert A.A.C., Vogels R., Custers J.H.H.V., Addo M.M. (2005). Neutralizing antibodies to adenovirus serotype 5 vaccine vectors are directed primarily against the adenovirus hexon protein. J. Immunol. Baltim. Md..

[36] Hong S.S., Habib N.A., Franqueville L., Jensen S., Boulanger P.A. (2003). Identification of adenovirus (ad) penton base neutralizing epitopes by use of sera from patients who had received conditionally replicative ad (addl1520) for treatment of liver tumors. J. Virol..

[37] Zak D.E., Andersen-Nissen E., Peterson E.R., Sato A., Hamilton M.K., Borgerding J. (2012). Merck Ad5/HIV induces broad innate immune activation that predicts CD8^+^ T-cell responses but is attenuated by preexisting Ad5 immunity. Proc. Natl. Acad. Sci. USA.

[38] Varnavski A.N., Zhang Y., Schnell M., Tazelaar J., Louboutin J.-P., Yu Q.-C. (2002). Preexisting immunity to adenovirus in rhesus monkeys fails to prevent vector-induced toxicity. J. Virol..

[39] Varnavski A.N., Calcedo R., Bove M., Gao G., Wilson J.M. (2005). Evaluation of toxicity from high-dose systemic administration of recombinant adenovirus vector in vector-naive and pre-immunized mice. Gene Ther..

[40] Perreau M., Pantaleo G., Kremer E.J. (2008). Activation of a dendritic cell-T cell axis by Ad5 immune complexes creates an improved environment for replication of HIV in T cells. J. Exp. Med..

[41] Cotter M.J., Zaiss A.K., Muruve D.A. (2005). Neutrophils interact with adenovirus vectors via Fc receptors and complement receptor 1. J. Virol..

[42] Zaiss A.K., Vilaysane A., Cotter M.J., Clark S.A., Meijndert H.C., Colarusso P. (2009). Antiviral antibodies target adenovirus to phagolysosomes and amplify the innate immune response. J. Immunol. Baltim. Md..

[43] Tang J., Olive M., Pulmanausahakul R., Schnell M., Flomenberg N., Eisenlohr L. (2006). Human CD8+ cytotoxic T cell responses to adenovirus capsid proteins. Virology.

[44] Sumida S.M., Truitt D.M., Kishko M.G., Arthur J.C., Jackson S.S., Gorgone D.A. (2004). Neutralizing Antibodies and CD8+ T Lymphocytes both Contribute to Immunity to Adenovirus Serotype 5 Vaccine Vectors. J. Virol..

[45] Olive M., Eisenlohr L., Flomenberg N., Hsu S., Flomenberg P. (2002). The adenovirus capsid protein hexon contains a highly conserved human CD4+ T-cell epitope. Hum. Gene Ther..

[46] Bett A.J., Prevec L., Graham F.L. (1993). Packaging capacity and stability of human adenovirus type 5 vectors. J. Virol..

[47] Parks R.J., Graham F.L. (1997). A helper-dependent system for adenovirus vector production helps define a lower limit for efficient DNA packaging. J. Virol..

[48] Parks R.J., Bramson J.L., Wan Y., Addison C.L., Graham F.L. (1999). Effects of Stuffer DNA on Transgene Expression from Helper-Dependent Adenovirus Vectors. J. Virol..

[49] Aderem A., Ulevitch R.J. (2000). Toll-like receptors in the induction of the innate immune response. Nature.

[50] Hoebe K., Janssen E., Beutler B. (2004). The interface between innate and adaptive immunity. Nat. Immunol..

[51] Kawai T., Akira S. (2006). Innate immune recognition of viral infection. Nat. Immunol..

[52] Ahmad-Nejad P., Häcker H., Rutz M., Bauer S., Vabulas R.M., Wagner H. (2002). Bacterial CpG-DNA and lipopolysaccharides activate Toll-like receptors at distinct cellular compartments. Eur. J. Immunol..

[53] Latz E., Schoenemeyer A., Visintin A., Fitzgerald K.A., Monks B.G., Knetter C.F. (2004). TLR9 signals after translocating from the ER to CpG DNA in the lysosome. Nat. Immunol..

[54] Cerullo V., Seiler M.P., Mane V., Brunetti-Pierri N., Clarke C., Bertin T.K. (2007). Toll-like receptor 9 triggers an innate immune response to helper-dependent adenoviral vectors. Mol. Ther. J. Am. Soc. Gene Ther..

[55] Kim I.H., Józkowicz A., Piedra P.A., Oka K., Chan L. (2001). Lifetime correction of genetic deficiency in mice with a single injection of helper-dependent adenoviral vector. Proc. Natl. Acad. Sci. USA.

[56] Toietta G., Mane V.P., Norona W.S., Finegold M.J., Ng P., McDonagh A.F. (2005). Lifelong elimination of hyperbilirubinemia in the Gunn rat with a single injection of helper-dependent adenoviral vector. Proc. Natl. Acad. Sci. USA.

[57] Morral N., O’Neal W., Rice K., Leland M., Kaplan J., Piedra P.A. (1999). Administration of helper-dependent adenoviral vectors and sequential delivery of different vector serotype for long-term liver-directed gene transfer in baboons. Proc. Natl. Acad. Sci. USA.

[58] Brunetti-Pierri N., Ng T., Iannitti D., Cioffi W., Stapleton G., Law M. (2013). Transgene expression up to 7 years in nonhuman primates following hepatic transduction with helper-dependent adenoviral vectors. Hum. Gene Ther..

[59] Muruve D.A., Cotter M.J., Zaiss A.K., White L.R., Liu Q., Chan T. (2004). Helper-Dependent Adenovirus Vectors Elicit Intact Innate but Attenuated Adaptive Host Immune Responses In Vivo. J. Virol..

[60] Delgado C., Francis G.E., Fisher D. (1992). The uses and properties of PEG-linked proteins. Crit. Rev. Ther. Drug Carr. Syst..

[61] Parveen S., Sahoo S.K. (2006). Nanomedicine: Clinical applications of polyethylene glycol conjugated proteins and drugs. Clin. Pharmacokinet..

[62] Fisher K.D., Stallwood Y., Green N.K., Ulbrich K., Mautner V., Seymour L.W. (2001). Polymer-coated adenovirus permits efficient retargeting and evades neutralising antibodies. Gene Ther..

[63] O’Riordan C.R., Lachapelle A., Delgado C., Parkes V., Wadsworth S.C., Smith A.E. (1999). PEGylation of adenovirus with retention of infectivity and protection from neutralizing antibody in vitro and in vivo. Hum. Gene Ther..

[64] Croyle M.A., Chirmule N., Zhang Y., Wilson J.M. (2001). Stealth adenoviruses blunt cell-mediated and humoral immune responses against the virus and allow for significant gene expression upon readministration in the lung. J. Virol..

[65] Croyle M.A., Chirmule N., Zhang Y., Wilson J.M. (2002). PEGylation of E1-deleted adenovirus vectors allows significant gene expression on readministration to liver. Hum. Gene Ther..

[66] Mok H., Palmer D.J., Ng P., Barry M.A. (2005). Evaluation of polyethylene glycol modification of first-generation and helper-dependent adenoviral vectors to reduce innate immune responses. Mol. Ther. J. Am. Soc. Gene Ther..

[67] Subr V., Kostka L., Selby-Milic T., Fisher K., Ulbrich K., Seymour L.W. (2009). Coating of adenovirus type 5 with polymers containing quaternary amines prevents binding to blood components. J. Control Release Off. J. Control Release Soc..

[68] Green N.K., Herbert C.W., Hale S.J., Hale A.B., Mautner V., Harkins R. (2004). Extended plasma circulation time and decreased toxicity of polymer-coated adenovirus. Gene Ther..

[69] Kreppel F., Gackowski J., Schmidt E., Kochanek S. (2005). Combined genetic and chemical capsid modifications enable flexible and efficient de- and retargeting of adenovirus vectors. Mol. Ther. J. Am. Soc. Gene Ther..

[70] Corjon S., Wortmann A., Engler T., van Rooijen N., Kochanek S., Kreppel F. (2008). Targeting of adenovirus vectors to the LRP receptor family with the high-affinity ligand RAP via combined genetic and chemical modification of the pIX capsomere. Mol. Ther. J. Am. Soc. Gene Ther..

[71] Prill J.-M., Espenlaub S., Samen U., Engler T., Schmidt E., Vetrini F. (2011). Modifications of adenovirus hexon allow for either hepatocyte detargeting or targeting with potential evasion from Kupffer cells. Mol. Ther. J. Am. Soc. Gene Ther..

[72] Krutzke L., Prill J.M., Engler T., Schmidt C.Q., Xu Z., Byrnes A.P. (2016). Substitution of blood coagulation factor X-binding to Ad5 by position-specific PEGylation: Preventing vector clearance and preserving infectivity. J. Control Release Off. J. Control Release Soc..

[73] Khare R., Reddy V.S., Nemerow G.R., Barry M.A. (2012). Identification of adenovirus serotype 5 hexon regions that interact with scavenger receptors. J. Virol..

[74] Prill J.-M., Subr V., Pasquarelli N., Engler T., Hoffmeister A., Kochanek S. (2014). Traceless bioresponsive shielding of adenovirus hexon with HPMA copolymers maintains transduction capacity in vitro and in vivo. PLoS ONE.

[75] Schmid M., Ernst P., Honegger A., Suomalainen M., Zimmermann M., Braun L. (2018). Adenoviral vector with shield and adapter increases tumor specificity and escapes liver and immune control. Nat. Commun..

[76] Campos S.K., Barry M.A. (2006). Comparison of adenovirus fiber, protein IX, and hexon capsomeres as scaffolds for vector purification and cell targeting. Virology.

[77] Espenlaub S., Corjon S., Engler T., Fella C., Ogris M., Wagner E. (2010). Capsomer-specific fluorescent labeling of adenoviral vector particles allows for detailed analysis of intracellular particle trafficking and the performance of bioresponsive bonds for vector capsid modifications. Hum. Gene Ther..

[78] Zhang C., Zhou D. (2016). Adenoviral vector-based strategies against infectious disease and cancer. Hum. Vaccines Immunother..

[79] Folegatti P.M., Ewer K.J., Aley P.K., Angus B., Becker S., Belij-Rammerstorfer S. (2020). Safety and immunogenicity of the ChAdOx1 nCoV-19 vaccine against SARS-CoV-2: A preliminary report of a phase 1/2, single-blind, randomised controlled trial. Lancet.

[80] Zhu F.-C., Guan X.-H., Li Y.-H., Huang J.-Y., Jiang T., Hou L.-H. (2020). Immunogenicity and safety of a recombinant adenovirus type-5-vectored COVID-19 vaccine in healthy adults aged 18 years or older: A randomised, double-blind, placebo-controlled, phase 2 trial. Lancet.

[81] Logunov D.Y., Dolzhikova I.V., Zubkova O.V., Tukhvatulin A.I., Shcheblyakov D.V., Dzharullaeva A.S. (2020). Safety and immunogenicity of an rAd26 and rAd5 vector-based heterologous prime-boost COVID-19 vaccine in two formulations: Two open, non-randomised phase 1/2 studies from Russia. Lancet Lond. Engl..

[82] Logunov D.Y., Dolzhikova I.V., Shcheblyakov D.V., Tukhvatulin A.I., Zubkova O.V., Dzharullaeva A.S. (2021). Safety and efficacy of an rAd26 and rAd5 vector-based heterologous prime-boost COVID-19 vaccine: An interim analysis of a randomised controlled phase 3 trial in Russia. Lancet Lond. Engl..

[83] Ramasamy M.N., Minassian A.M., Ewer K.J., Flaxman A.L., Folegatti P.M., Owens D.R. (2021). Safety and immunogenicity of ChAdOx1 nCoV-19 vaccine administered in a prime-boost regimen in young and old adults (COV002): A single-blind, randomised, controlled, phase 2/3 trial. Lancet Lond. Engl..

[84] Tripathy S.K., Black H.B., Goldwasser E., Leiden J.M. (1996). Immune responses to transgene-encoded proteins limit the stability of gene expression after injection of replication-defective adenovirus vectors. Nat. Med..

[85] Yang Y., Su Q., Wilson J.M. (1996). Role of viral antigens in destructive cellular immune responses to adenovirus vector-transduced cells in mouse lungs. J. Virol..

[86] Harui A., Roth M.D., Kiertscher S.M., Mitani K., Basak S.K. (2004). Vaccination with helper-dependent adenovirus enhances the generation of transgene-specific CTL. Gene Ther..

[87] Schirmbeck R., Reimann J., Kochanek S., Kreppel F. (2008). The immunogenicity of adenovirus vectors limits the multispecificity of CD8 T-cell responses to vector-encoded transgenic antigens. Mol. Ther. J. Am. Soc. Gene Ther..

[88] Weaver E.A., Nehete P.N., Buchl S.S., Senac J.S., Palmer D., Ng P. (2009). Comparison of Replication-Competent, First Generation, and Helper-Dependent Adenoviral Vaccines. PLoS ONE.

[89] Kron M.W., Engler T., Schmidt E., Schirmbeck R., Kochanek S., Kreppel F. (2011). High-capacity adenoviral vectors circumvent the limitations of ΔE1 and ΔE1/ΔE3 adenovirus vectors to induce multispecific transgene product-directed CD8 T-cell responses. J. Gene Med..

[90] Wortmann A., Vöhringer S., Engler T., Corjon S., Schirmbeck R., Reimann J. (2008). Fully detargeted polyethylene glycol-coated adenovirus vectors are potent genetic vaccines and escape from pre-existing anti-adenovirus antibodies. Mol. Ther. J. Am. Soc. Gene Ther..

[91] Weaver E.A., Barry M.A. (2008). Effects of Shielding Adenoviral Vectors with Polyethylene Glycol on Vector-Specific and Vaccine-Mediated Immune Responses. Hum. Gene Ther..

